# Sustaining and expanding telehealth activity: Training requirements for Australian residential aged care front-line staff

**DOI:** 10.1016/j.pecinn.2022.100109

**Published:** 2022-11-28

**Authors:** Annie Banbury, Monica L. Taylor, Leonard C. Gray, Natasha Reid, Anthony C. Smith

**Affiliations:** a Centre for Online Health, Centre for Health Services Research, The University of Queensland, Ground Floor, Building 33, Princess Alexandra Hospital, 199 Ipswich Road, Woolloongabba, QLD 4102, Australia; b Centre for Health Services Research, The University of Queensland, Level 2, Building 33, Princess Alexandra Hospital, 199 Ipswich Road, Woolloongabba, QLD 4102, Australia; c Centre for Innovative Medical Technology, University of Southern Denmark, Odense University Hospital, Kløvervænget 8C, entrance 101, 3. Floor, DK-5000 Odense C, Denmark

**Keywords:** Residential aged care, Telehealth, Telemedicine, Training, Nursing home

## Abstract

**Objective:**

To identify the training needs of front-line aged care staff as perceived by senior clinicians and managers at selected residential aged care facilities (RACFs).

**Methods:**

A qualitative explorative designed study using semi-structured interviews with a convenience sample of RACF senior managers and nurses. A hybrid analysis approach using a framework deductive analysis followed by inductive analysis for sub-themes.

**Results:**

Four sub-themes emerged to sustain increased telehealth activity: technology knowledge and digital literacy skills, including understanding telehealth ecosystems and technical skills; evidence-based reviews and clinical frameworks for telehealth consultations to identify appropriate consultations and successful use cases; telehealth best practice guidelines and workflows including telehealth consultations protocols, communicating by videoconferencing, how to support families in attending telehealth consultations and optimal training models; and telehealth policy and legal guidance.

**Conclusion:**

Staff require comprehensive training to sustain and expand telehealth use in RACFs. Training should focus on knowledge, skills and competencies in using telehealth as well as the broad factors of policies and understanding ICT systems to support staffs’ abilities and confidence.

**Innovation:**

This study provides innovative findings that identify key components and associated activities and resources for training RACF staff to ensure they have sufficient knowledge, competency, skills and confidence to integrate telehealth into care provision.

## Highlights

• Data were extracted deductively to the Telehealth Program Pathway Framework.
• Staff reported videoconferencing was underused in RACFs and had future value.
• RACFs should control video links to avoid using multiple health provider systems.
• Beyond being tech-savvy, staff should know the value of telehealth and workflows.
• We provide a blueprint on key components for a RACF telehealth training program.

## Graphical abstract

**Figure:**
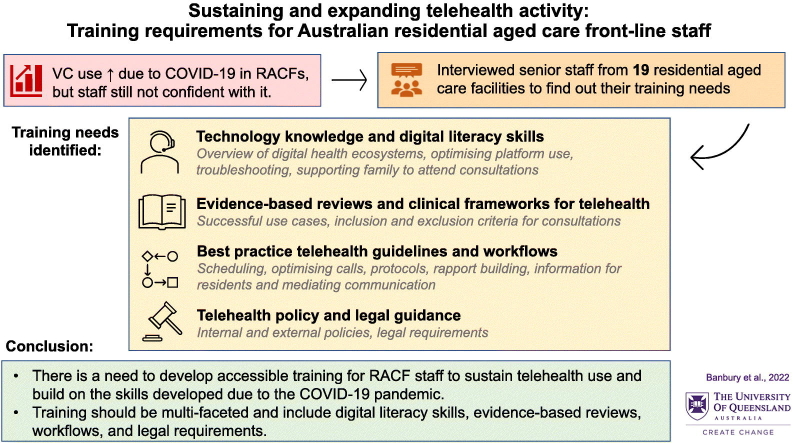

## 1 Introduction

The coronavirus disease 2019 (COVID-19) pandemic has resulted in the unprecedented uptake of telehealth across all healthcare sectors [1]. In Australia, telehealth funding changes have made it more appealing to general and specialist health practitioners to conduct telehealth consultations with their patients [2]. The increasing adoption of telehealth during the pandemic has encouraged changes in how health services are delivered and recognition of critical factors contributing to telehealth integration and sustainability [[3], [4], [5], [6], [7]]**.** To promote the uptake of telehealth, we need a trained workforce who understand telehealth and have the skills to practice telehealth [3].

In September 2020, an Australian Government-led Royal Commission into Aged Care Quality and Safetypublished a Special Report on Aged Care and COVID-19 [8]. The report listed several recommendations to the government regarding the use of telehealth in aged care. These include expanding access to subsidised specialist telehealth services; ensuring each resident is enrolled with a General Practitioner (GP),and there is agreement on how care is provided, including the use of telehealth services; and there is appropriate information and communications technology (ICT) infrastructure and investment in technology.

The Royal Commission acknowledged the importance of training for aged care staff, including minimum qualification needs, upskilling staff to ensure they can competently perform nursing tasks, and ensuring that staff have the capability to support telehealth services. From a nursing perspective, telehealth training is required to ensure staff have the necessary knowledge and skills. Digital literacy skills, defined as capabilities for living, working and participating in a digital society [9] are becoming increasingly important forhealthcare providers. However, there is a lack of published data on the digital literacy knowledge and skills [10] and training requirements of residential aged care nursing staff [11].

### 1.1 Aim

This study aimed to assess the telehealth training needs of front-line aged care staff, according to feedback provided by senior clinicians and managers at selected residential aged care facilities (RACFs).

## 2 Methods

### 2.1 Design and setting

A qualitative, exploratory designed study to investigate the experiences of RACFs in using telehealth during the pandemic. Between April and May 2020, at the start of COVID-19, training was provided by Zoom for senior managers and their nominated nursing staff at 53 residential aged care facilities within Brisbane, Australia. The training lasted for 20 minutes and demonstrated how to connect to the state-wide Queensland Health Telehealth Portal to access Queensland Health services.

### 2.2 Participants

We compiled a list of facilities and clinical leads who attended the training, which formed our convenience sample. Where multiple RACFs were owned by one organisation, one facility was invited to take part in the study.

### 2.3 Ethics

Ethical approval was obtained from The University of Queensland Human Research Ethics Committee (Approval No. 2020002172). All participants gave consent to participate in the study.

### 2.4 Recruitment and data collection

In September 2020, clinical contact leads from sample sites were invited to participate in the study. Study information brochures and consent forms were sent by e-mail to 39 key contacts at the RACFs. Researchers followed the email by telephone to organise a semi-structured videoconference or telephone interview. Research questions focused on service design, the technology used, adoption and engagement, outcomes and impact on staff and residents and how to sustain telehealth activity. Researchers (AB, MT and NR) experienced in telehealth and/or ageing research conducted three test interviews and made minor amendments to the questions after a group discussion. Interviews were recorded using Zoom and automated transcriptions were generated and cleaned by checking their accuracy against the recordings. Interviews lasted approximately 45 minutes, and data were collected until saturation, when no new themes were identified.

### 2.5 Data analysis

We employed a hybrid approach of qualitative thematic analysis methods [12] using NVivo [13]. The Telehealth Program Pathway framework [14] (Table 1) was used to analyse RACFs' telehealth models, perceptions of healthcare providers' and residents' engagement, and perceptions of telehealth activity and how it could be sustained. Firstly, transcripts were read through; using a deductive approach [14], data were extracted to all relevant framework components by AB, i.e. data could be coded to more than one component and then reviewed by MT. Second stage inductive coding by AB and MT thematically identified sub-themes within each component of The Telehealth Program Pathway, which we amended to include the additional component "Sustaining telehealth activity". The sub-themes were regularly shared with all team members for peer discussion, refinement and agreement. This study reports participants' perceptions of their staff's training needs to sustain telehealth activity. To provide context, we briefly report on the extent of telehealth adoption by RACF in response to COVID-19.

**Table 1:** Telehealth pathway framework (Agboola 2014).

Telehealth component	Description
Service design	Residential aged care facility and residents' characteristics and external care providers
Technology & operations	Resources required to deliver telehealth activity including hardware, software, Internet connectivity, personnel, referral process and patient flow
Adoption & engagement	Actions required for telehealth uptake (organisational readiness, specialty use, ease and willingness of use, barriers/challenges, facilitators/enablers, workflow integration)
Outcomes & impact on organisations, staff and residents	Perception of telehealth activity on intermediate outcomes and overall impact on staff, residents and the organisation (capacity to engage, satisfaction, workload changes in role)
Sustaining telehealth activity	Resources and support required for future telehealth use

## 3 Results

### 3.1 Participants

Of the 39 invited subjects, 19 staff (49%) agreed to participate in the study. Reporting to be ‘too busy’ was the most common reason provided by participants who declined to take part. Participants represented 19 different RACFs, and most (90%, *n* = 17) were responsible for clinical care (Directors of Nursing, Clinical Care Consultants, Facility Managers). Two participants (10%, *n* = 2) were registered nurses without senior administrative responsibilities. All participants were able to advise on their facilities' use of telehealth and their perceived training needs. Eleven facilities were not-for-profit and on average, there were 87 beds per facility (range 42–140).

### 3.2 Telehealth adoption

All facilities reported an increase in telehealth activity by telephone and video. In general, the telephone was predominantly used for telehealth consultations. However, staff reported that videoconferencing remained underutilised in their facility and this consultation mode had potential value for future aged care service delivery. Before the COVID-19 restrictions, videoconsultations were limited or non-existent in RACF settings. Staff were comfortable using the telephone for consultations before COVID-19 but less familiar with videoconferencing for clinical consultations. Although staff have now been upskilled, some are still not confident in the use of videoconferencing.

“The [digital] literacy of using the technology, both for the staff and the residents [before the pandemic], was that not all staff knew how to use Skype or Zoom. Now probably 80% are okay, but there's still the 20% that's not really that confident to use it.” [P14]

### 3.3 Training requirements

**Fig. 1:**
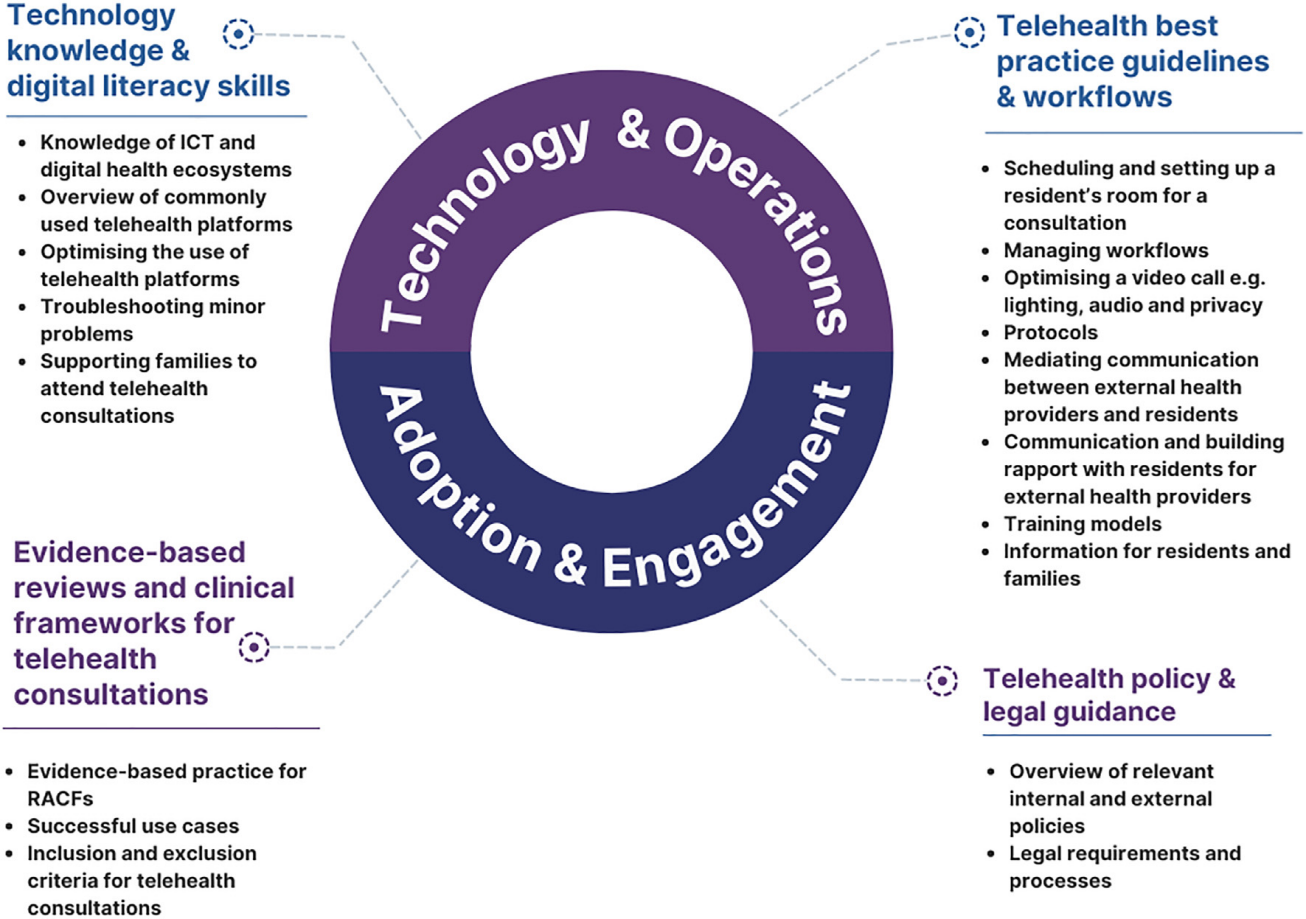
Training requirements to sustain telehealth activity in residential aged care facilities.

Four sub-themes emerged from the "Sustaining telehealth activity" framework component, which described RACF managers' and senior nursing staffs' perceptions about their teams' training needs to sustain and improve video consultations. These were as follows: (1) Technology knowledge and digital literacy skills; (2) Evidence-based reviews and clinical frameworks for telehealth consultations; (3) Telehealth best practice guidelines and workflows; and (4) Telehealth policy and legal guidance. Fig. 1 outlines the themes and key factors.

#### 3.3.1 Technology knowledge and digital literacy skills

Participants reported staffhave differing levels of digital literacy skills and competencies. Most reported their staff could send text messages and browse the internet but lacked greater knowledge of ICT systems. In addition, some staff, particularly those trained overseas, are unfamiliar with technology and lacked the confidence and skills to use it within the workplace.

“We have staff who are not familiar with tech, they talk and text on the phone but that's about it and it's very scary for these staff to actually use the technology.” [P19]

A lack of knowledge and understanding about how ICT systems are configured and operated was reported. It is worth noting that several participants could not access the Zoom link sent for the interviews; rather, they connected using the telephone. Firewalls, connection issues, and difficulties with logging in to multiple systems were all reported with little understanding of how to overcome these issues.

“Our firewall policy would block a lot of meetings from different parties. Even for the Zoom meeting, it's failed twice. It keeps popping up a window saying the policy will not allow you to keep connecting. I think that's part of the setup of computers here for confidentiality - we probably need more training I suppose to work through all these things.” [P6]

“When we have a meeting, it just keeps downloading. Oh, I don't know what it does, it's going in a loop. And it was not opening so I don't know what the problem was. Then finally, they told me that my Explorer was not updated. I don't know the technical term for that - it wasn't working.” [P1]

RACF staff received video consultation invites from external healthcare providers using several different platforms. They had to use and troubleshoot multiple platforms, across multiple devices until they found a method that worked.

“The [practitioner] was doing Healthdirect, I think, and Zoom and then sometimes they would be directly calling …. But if it doesn't work and we are finding it drops out, then occasionally one or two times we used FaceTime.” [P16]

Participants perceived that telehealth systems have more capabilities to enhance their consultations than they were aware of. Therefore, training for optimising the use of platforms to their full extent would be beneficial.

“Training should be focused on getting the best out of the systems, really understanding how they work and their best use, managing the workflow – those kinds of issues.” [P19]

At times staff were required to support residents' family members using digital devices to attend clinical consultations or socially connect with residents. A staff member with higher levels of digital literacy skills fulfilled this role for clinical video calls, and diversional therapists supported social calls for those who employed them.

#### 3.3.2 Evidence-based reviews and clinical frameworks for telehealth consultations

Participants identified they required evidence-based information on using telehealth in the aged care setting. They wanted information on the effectiveness of video telehealth compared to in-person consultations for the most common types of clinical consultations and inclusion and exclusion criteria, in other words, when it is and is not appropriate to undertake a video telehealth consultation. It was suggested real-world case studies that detail how telehealth has been successfully deployed and integrated into everyday care would be of high interest and help managers understand suitable models.

#### 3.3.3 Telehealth best practice guidelines and workflows

Best practice guidelines that encourage the routine delivery of clinically appropriate and high-quality telehealth services are needed. Guidelines should cover issues such as preparation, room configuration, privacy, audio and lighting. In addition, guidance for mediating conversations and aiding health providers in eliciting information from residents and how to position residents for health providers to be able to examine the resident is required. Participants were acutely aware of the limited time health providers had to spend with residents, and they wanted to optimise the time as much as possible.

“Ongoing education in the future would be about setting up for the appointment. It is a clinical appointment not just your casual FaceTiming and social interaction that are made as telehealth appointments. They need to consider privacy, positioning everybody, the lighting and the sound, especially for our residents that do struggle with their hearing and vision. You don't want to spend half the telehealth time adjusting all those things, and not actually talking or dealing with the issues that you were calling through for.” [P7]

“If you're going to use telehealth, you need to be taught all the facets so you have the methods to get the best out of it. We don't want to waste a specialist's time and they don't know what they saw or we didn't answer the questions to help build the picture.” [P4]

Clinicians providing consultations would also benefit from training and best practice guidance in factors such as building rapport and undertaking clinical assessments with aged care residents. For successful telehealth consultations, resourcing requirements such as additional staff and/or equipment should be identified before the start of the consultation.

“The doctor got a bit stroppy when they couldn't immediately answer all the questions, or the resident wouldn't sit up exactly like the doctor wanted them to do. But the staff member was in the room by themselves with the door closed, etc. And it was, give us chance, if it's an eighty-year-old lady who actually normally needs two people to be assisted, I'm not going to be able to do it by myself. So perhaps a little bit of understanding from the doctors around what, what could be done.” [P10]

Nursing staff require more education to encourage them to consider a telehealth appointment for residents rather than always booking an in-person appointment.

“It's a bit about reprogramming the thinking of the Registered Nurse around, okay, Mrs. Brown has got this appointment, it's going to be really difficult for her to get there, her family can't take her, how about we try and set up a telehealth appointment?" [P13]

RACFs have a high turnover of staff, therefore training should be regular and ongoing to ensure new staff members are fully trained. Participants acknowledged that conducting telehealth consultations requires a skill set that could be easily learned, however, confidence and competence require practical experience. Once trained, staff need to use telehealth regularly otherwise, the knowledge is easily forgotten.

“Nothing's any good without good training. I attended great training and information on psychotropic drugs and I was listening, listening, listening and then two weeks later I went to use it. And it was like blank-o.” [P4]

Several RACFs had only trained their registered nurses and some of their enrolled nurses (EN) to use telehealth. However, they recognised a need to extend training to auxiliary nurses and carers.

“From an auxiliary nurse or a carer perspective if we want them to use it to some degree then we really needed all to do some more training. But at the moment, we've just done it from our ENs and RNs, kind of upwards, because we feel that if there's going to be a clinical question asked, then those are the people who may be able to answer them better than an AIN or a carer” [P2]

For residents who can take part in a video consultation, information that explains what a telehealth consultation is and how it compares to an in-person consultation would be beneficial. Residents may not always realise they have participated in a clinical consultation because the health practitioner has not visited in person. Information should be available in formats that are suitable for older people. It was suggested this could be in large print, audio format or information that staff read to them.

“I think a simplified education for residents as well. They deserve to have the privacy of discussing something with a specialist without us in the room. I think that could be in either audible form or large print they can read, it's been a huge benefit for them.” [P18]

“I think we need education on how residents get the best out of it [telehealth] as well. We have people who are cognitively impaired but they're not zero. They're used to seeing and chatting to the doctor. Telehealth can be impersonal for the elderly - it's like they haven't really been to the doctor. I think you need to develop skills with the people delivering the telehealth so that they [older people] don't feel disconnected and feel they have to look at a screen because the doctor doesn't want them to visit.” [P4]

RACFs deployed differing training models for upskilling staff in using telehealth platforms. Peer education was most commonly used in organisations where at least one member of staff had good digital literacy skills. They were tasked with organising video consultations and training their colleagues.

“Some of the staff were a little hesitant, those that are not as IT skilled. But once they were shown and given a bit of education and support, they were more than happy to go ahead with it and realised how easy it was.” [P7]

Other training models included using external healthcare providers who delivered services by telehealth, cascade training and small group training. Best practice guidelines should consist of training models that are most successful for RACFs, including those with high staff turnover rates and high use of temporary agency staff. In addition, training should be ongoing and provide updates on changes to platforms or health providers who were willing to take part in video consultations.

“A 6 to 12 monthly update with news of any of the new technology, any changes to the use of different platforms or different services.” [P18]

In addition, family members would benefit from information explaining how telehealth can be used with older people and the types of consultations for which it is effective.

#### 3.3.4 Telehealth policy and legal issues

RACFs reported regularly using fax and emails to communicate with GPs for changes in residents' care plans, medications and other documents. However, some participants reported that signatures for these documents need to be originals and thought electronic signatures were not permissible.

“For us it's getting original signatures on things. If you're getting a signature via fax or an email, you really have to block that out and run that [documentation] up to get an original signature. Otherwise, the Commission doesn't see it as being signed - you've only got a 48-hour period… legislation would have to change so that those types of electronic signatures became a legal document.” [P1]

## 4 Discussion and conclusion

### 4.1 Discussion

This study reinforces the importance of telehealth training for RACF staff as a critical requirement for the uptake of telehealth in these settings. It reports the training needs of front-line aged care staff to utilize and sustain telehealth activity as perceived by senior clinicians and managers at RACFs. Our findings have identified that although telehealth was rapidly adopted during the pandemic, to build and sustain this activity, staff require training that is broader than just being able to use a telehealth platform. Training must improve digital health literacy capabilities and practices supported by a range of evidence-based resources. Our findings are similar to others who have highlighted inadequate training of RACF staff and other healthcare professionals to use telehealth competently and confidently [15,16].

RACF training should focus on technical knowledge, skills and competencies [3,10,17], best practice guidelines and optimum workflows on how to implement telehealth into routine care successfully [3], clinical frameworks and evidence-based reviews on the efficacy of telehealth for older people and an understanding of policy and legal issues when using telehealth [18].

Significant workforce issues pertaining to RACFs include high staff turnover, a heavy reliance on agency nursing staff, and a lack of digital literacy and confidence [19,20]. Similar to findings from Rainsford [21], our study found that RACF staff during the pandemic had rapidly transitioned to using telehealth with little time for training. Telehealth could support more effective health care post-COVID-19, however, if current usage is not supported or sustained, there will be missed opportunities and hard-won skills developed throughout the pandemic will be lost. To sustain telehealth's use for RACF staff, health professionals providing care and residents require ongoing, context-specific education and training [[22], [23], [24], [25]].

Results from this study indicate that many staff who were required to take part in telehealth consultations had limited knowledge about how ICT systems work or the ability to troubleshoot minor technical issues. This is not surprising since nursing is primarily a hands-on profession. However, this lack of IT knowledge can lead to frustration and potential non-adoption of technology for future consultations. The Australian government has indicated that technology will have a key role in improving and reforming the aged care sector in response to The Royal Commission into Aged Care Quality and Safety's final report [8]. The Final Report included recommendations to implement digital care management systems and electronic medication management. Therefore, it is increasingly important for staff to understand information technology systems and how they can interconnect information and care mechanisms, such as telehealth, to function as a unit in a digital ecosystem.

Training should ensure staff have sufficient digital literacy for rudimentary troubleshooting and a basic understanding of digital health ecosystems to ensure sustained use. RACFs navigate multiple telehealth systems because health providers generate the video call link. It would be beneficial for RACFs to take more control of how and when consultations take place by generating telehealth consultation links using familiar systems. Furthermore, this would give them greater control of their telehealth workflows and appointment scheduling for easier integration into their workflows, avoiding busy times or when residents are less likely to engage with the clinician.

Senior RACF staff identified they wanted to ensure staff were able to support residents and health practitioners in efficient, high-quality consultations. They understand health consultations differ from social interactions by video call, and further guidance such as telehealth best practice guidelines, evidence reviews and clinical frameworks are required. Training should include an explanation of the value proposition and protocols for using telehealth [15,26] so that staff appraise whether a telehealth consultation can address the medical needs of a resident. Telehealth skills training is also required to ensure health providers have the best possible vision and audio to optimise a consultation. Developing specific telehealth nursing competencies, such as performing physical examinations to support health providers, is warranted [23,27]. However, there is a lack of research on telenursing skills, particularly within RACFs [27]. Our results indicated there can be frustration from external health providers when using telehealth with RACF staff. To help overcome this, it would be beneficial for external clinicians to take part in training to support rapport-building and understanding of the clinical modifications for telehealth consultations [28].

Our study highlights that RACFs need to consider how telehealth training is delivered. Many RACFs reported using peer training, relying on one staff member with higher levels of digital literacy. Although peer training is likely to be consistent, this model can create bottlenecks with staff having to wait to access training. Online training has been suggested as the most accessible, flexible and effective method for RACFs because of staff shortages and health workloads [29]. However, ‘buy-in’ from RACF managers is required for it to be successful [30]. Developing national training for generic skills and competencies would ensure quality, consistency and equitable access for all RACFs. However, technical training is dependent on RACFs' telehealth systems. Training should become part of onboarding activities for new staff, with regular updates and be ongoing to accommodate frequent staff changes [31]. Our participants suggested training should be every 6–12 months, however, frequent, intensive training for staff who are responsible for telehealth consults can help in identifying early changes in residents' conditions that can be attended to in a telehealth consultation, which may prevent a hospital transfer [32].

Telehealth training throughout the health sector is required to sustain telehealth's increased use since the pandemic. Although we highlight the importance of telehealth training for staff in RACFs, health professionals in all settings have a role in providing or receiving telehealth consultations**.** Our study has identified knowledge and skills that should be included in telehealth training for RACF staff. However; a successful telehealth service requires not only an effective training program but also other vital factors, such as funding for new models of care, fit-for-purpose technology, a willingness to change and an acceptance of new ways of working [3]. Telehealth will only work seamlessly if health providers are well-trained and part of a health system that supports its use.

This study has some limitations. The data were collected as part of a larger project to evaluate the experiences of RACF staff in using telehealth during COVID-19 restrictions, not for the sole purpose of identifying staff training needs. This smaller study is focused on how we ensure we optimise and further extend the telehealth skills front-line staff rapidly learnt during the pandemic. There may be some selection bias among those who agreed to participate, with those having more positive experiences or taking part in training more willing to participate in the study. However, participants were encouraged to share diverse telehealth experiences and the training needs to sustain and expand telehealth activity. The research team was not involved with any prior training. Participants were from a metropolitan area, and experiences and perceptions may not reflect the perceptions of RACF in other geographical locations. Although participants were evenly spread across for-profit and not-for-profit organisations, it is possible that rurally located RACF staff may have different training needs, and we recommend further research to understand these needs. Two interviewers are experienced telehealth researchers which may introduce bias, however, to minimise this, the researcher (NR) without telehealth experience was invited to collaborate.

### 4.2 Innovation

This study is focused on innovative care practices using telehealth in RACFs. It identifies the critical components of an innovative training program for RACF staff, as perceived by RACF managers and front-line staff responsible for training. In particular, it highlights the importance of staff having a broader knowledge of ICT and telehealth ecosystems, which will help them to understand issues such as how data is transmitted, where it is stored and the technical skills required to troubleshoot minor problems. In addition, broader contextual knowledge should be provided on policy and legal guidance. The study highlights the opportunity to use multi-point telehealth with residents, family members and clinicians. Multi-point consultations can support shared-decision making and ensure family members are kept informed of their loved one's care plans, potentially mitigating communication issues. This may require RACF staff to train family members to use telehealth. We also identify that residents and family members should be provided with appropriate information to support their understanding of telehealth and its effectiveness for care.

### 4.3 Conclusion

The results of our study are consistent with the recommendations published in the Aged Care Commission report – indicating an urgent need to develop accessible training for residential aged care staff to sustain telehealth use and build on the skills developed due to the COVID-19 pandemic. Telehealth training should be multi-faceted and include technical knowledge and skills, clinical frameworks, best practice guidelines and an understanding of policy and legal issues to meet the needs of RACF staff.

## Funding

This study was part of a broader study commissioned and funded by The Australian Partnership for Preparedness Research on Infectious Diseases Emergencies (APPRISE) Centre for Research Excellence.

## Declaration of Competing Interest

The authors declare that they have no known competing financial interests or personal relationships that could have appeared to influence the work reported in this paper.
